# Hydrogels in Regenerative Medicine and Other Biomedical Applications

**DOI:** 10.3390/ijms23063270

**Published:** 2022-03-18

**Authors:** Peter J. Jervis

**Affiliations:** Centre of Chemistry, Campus de Gualtar, University of Minho, 4710-057 Braga, Portugal; peterjervis@quimica.uminho.pt

It is my great pleasure to be part of this Special Issue in the *International Journal of Molecular Sciences*—“Hydrogels in Regenerative Medicine and Other Biomedical Applications”. Hydrogels are made up of three-dimensional matrices of hydrophilic polymers, which are held together by chemical or physical crosslinks or by supramolecular assemblies of smaller amphiphilic molecules. The gelation process can be initiated by a range of stimuli, including temperature, pH, electric or magnetic field, enzymatic modification, light, and others. Remarkably, these materials mainly consist of water molecules, in some cases containing over one million water molecules for every molecule of gelator. This unique class of materials represents a vast research field, owing to their many biomedical applications. These include uses in tissue engineering, 3D scaffolds for cell culture, biosensors, contact lenses, vehicles for controlled drug delivery, and many others. The focus of this Special Issue is related to regenerative medicine, an area that has witnessed rapid developments in recent years. Although much has already been discovered in this area, we continue to see excellent novel research, as exemplified by the seventeen manuscripts presented in this Special Issue, fourteen of which are original research articles.

A common theme in this Special Issue is the use of naturally occurring compounds and polymers for tissue engineering and wound-healing applications. For example, the group of Kang have improved the properties of fibrogen hydrogels by creating conjugates with keratin obtained from human hair. These new materials showed improved porosity. The keratin–fibrogen hydrogel also improved the proliferation of encapsulated human gingival fibroblasts (HGFs), which displayed a diffuse morphology when encapsulated by the new hydrogel [1]. Moon et al. report the development of hydrogels of triclosan (TCS)-complexed beta-cyclodextrin(β-CD)-conjugated methacrylated glycol chitosan (MGC) as antibacterial tissue adhesives. The hydrogel was formed through irradiation with UV light, and the in vitro and in vivo results reveal that the new hydrogel is able to inhibit bacterial infection and improve wound healing, showing its potential as a tissue bio-adhesive [2]. Tamer et al. used marjoram essential oil and kaolin to improve the properties of polyvinyl alcohol (PVA) sponges and created materials suitable for treating irregular wounds. Pore size, swelling capacity, antibacterial and antioxidant properties, and non-toxicity to fibroblasts were all improved, indicating the potential of the PVA/marjoram/kaolin sponge for hemostatic and antibacterial wound treatment [3]. Lin and Tang have described nanocomposites based on hydroxylapatite (HA), a naturally occurring mineral form of calcium apatite with the formula Ca_5_(PO_4_)_3_(OH). Combining cobalt complexes of this material with PVA produced a hydrogel (PVA–CoHA) that improved the mechanical and antibacterial properties over hydrogels made up of solely PVA. Furthermore, the local release of cobalt ions improved cell growth, thus showing potential for wound healing applications in the context of diabetic foot ulcers [4]. The group of Brun produced chitosan conjugated to cell adhesion peptide motifs (RGD and HVP). Their chitosan–RGD and chitosan–HVP conjugates were evaluated for their potential in bone tissue engineering. The optimal biomimetic properties were obtained using a 1:1 chitosan/chitosan–HVP system, which was superior to chitosan alone and chitosan—RGD systems, preserving the antibacterial effect of chitosan and supporting osteoblast cell adhesion [5].

Another important application of hydrogels is controlled drug delivery. García-Couce et al. have developed a novel strategy for the controlled release of betamethasone from AlgNa-g-poly(QCL-co-HEMA) hydrogels, allowing a sustained drug release over 8 hours. Furthermore, the new material was biocompatible with human chondrocytes and fibroblasts, which could be cultured on the hydrogels [6]. Additionally, related to cell culture, Svozilová et al. have created superporous poly(2-hydroxyethyl methacrylate-co-2-aminoethyl methacrylate) (P(HEMA-AEMA)) hydrogel scaffolds for in vitro 3D culturing of leukemic B cells. Modification with the RGDS cell-adhesion motif improved the survival of B-CLLs in cell culture, by promoting the cell–cell and cell–surface interactions. This model has potential for improving the screening of leukemia drugs, supplementing the use of in vivo animal protocols through the early exclusion of toxic and poorly active candidates [7].

Kim et al. present a proof-of-concept study where a hydrogel formed from methacrylated gelatin co-polymerized with a synthetic heparin mimic (GelMA-PSS) was able to serve as an in vitro platform to recapitulate in vivo muscle atrophy-like phenotypes. This was confirmed by the downregulation of key signaling pathways in cells cultured on the heparin-mimicking matrix. This in vitro platform may serve as a promising model of muscle injury model for drug screening and toxicity testing [8]. Safakas et al. have developed a hydrogel system with potential cell transplantation potential. Their NaALG-g-P(NIPAM80-co-NtBAM20)/DMEM formulation showed excellent shear-induced injectability at room temperature and instantaneous thermo-induced gel stiffening at body temperature. The rheological properties of this formulation are similar to those previously reported as ideal for cell transplantation applications. The biocompatibility of PNIPAM-based grafted polysaccharides makes this hydrogel a good candidate for further investigation [9].

Hydrogels continue to be developed as scaffolds related to 3D-bioprinting. Lee et al. used dual-nozzle, three-dimensional printing to develop adipose tissue-derived decellurized extracellular matrix (dECM) hydrogels based on highly elastic poly (L-lactide-co-ε-caprolactone) (PLCL). The 3D-printing technique used for the scaffold fabrication can be used to produce patient-specific scaffolds for later clinical application. The highly elastic PLCL co-polymer used in this study possesses physical properties closer to those of native adipose tissue than other polymers studied. The adECM hydrogel promotes adipose tissue reconstruction by encouraging neovascularization and tissue formation. Taken together, these materials could represent an alternative method for adipose tissue engineering [10]. Setayeshmehr et al. report novel, cell-compatible, dual-component, biomaterial inks, and bio-inks based on PVA and solubilized decellularized cartilage matrix (SDCM) hydrogels that can be utilized for cartilage bioprinting. Functionalizing PVA with cis-5-norbornene-endo-2,3-dicarboxylic anhydride (Nb) gave better results than functionalizing PVA with amine groups. The results show that the incorporation of SDCM into PVA-Nb reduces the compression modulus, enhances cell viability and bioprintability, and modulates the swelling ratio of the resulting hydrogels. Overall, PVA-Nb hydrogels containing SDCM show promise as versatile bio-inks for cartilage bioprinting [11].

Jin and Neogi present ultrasound imaging using a thermally tunable solid-state phononic crystal lens (TSSL). The results demonstrate the premise of applying a hydrogel-filled tunable solid-state lens in frequency domain echo-intensity modes and temporal domain time-of-flight modes, for monostatic detections and mappings in simple experimental environments. This polymer-based TSSL system presented improved tuneability in its focal length and far-field detection capability, compared with the water-based focusing sonic lens (FSL) of the same shape. This work represents the first practical experimental study of a thermally tunable solid-state phononic crystal focusing lens prototype [12].

In a study related to fertility restoration in men following cancer treatment, Del Vento et al. have used hydrogels supplemented with vascular growth factor-loaded nanoparticles in the transplantation of testicular tissue, ultimately leading to accelerated and improved vascular maturity. Poly(D,L-lactide-co-glycolide)/poly(ethylene glycol (PLGA/PLGA–PEG) was used to form nanoparticles containing vascular growth factors VEGF or PDGF. Each molecule was encapsulated individually in separate nanoparticles that were then combined within the alginate hydrogel. Supplementation of alginate hydrogels with nanoparticles delivering PDGF induced improvement in terms of vascularization and vascular maturity in testicular tissue grafts compared to supplementation with VEGF alone [13].

Hsiao et al. have developed a novel entrapment neuropathy model induced by ultrasound-guided perineural hydrogel injections. In entrapment neuropathy, a nerve becomes compressed between two other structures in the body (e.g., ligament and bone). Repetitive motion causes the ligament and bone to press or rub against the nerve, ultimately leading to numbness, tingling, burning, and weakness in the extremities. Inducing the condition in rats provides a surgery-free, minimally invasive animal model of entrapment neuropathy that could serve as a versatile tool in the search for effective treatments of this disease [14].

This Special Issue also contains three review articles, which we hope will provide excellent information resources for the hydrogel research community. Maji and Lee have provided an overview of the use of hydrogels as materials for developing three-dimensional in vitro models [15], while Alven and Aderibigbe have reviewed the use of hydrogels based on chitosan and cellulose and their applications in wound management [16]. Both of these reviews outline the outstanding progress being made in these respective areas. Finally, I had the pleasure of contributing a review from my own research team, whose research focuses on supramolecular peptide hydrogels. Compiled in collaboration with my esteemed colleagues Paula MT Ferreira and Jose Alberto Martins, we have published the first review of the literature specifically related to use of hydrogelators constructed from dehydropeptides, which are showing great promise for biomedical applications [17].
